# The Association Between Supragingival Plaque Microbial Profiles and the Clinical Severity of Oral Lichen Planus Subtypes: A Cross-Sectional Case–Control Study

**DOI:** 10.3390/jcm14145078

**Published:** 2025-07-17

**Authors:** Soo-Min Ok, Hye-Min Ju, Sung-Hee Jeong, Yong-Woo Ahn, Ji-Young Joo, Jung Hwa Park, Si Yeong Kim, Jin Chung, Hee Sam Na

**Affiliations:** 1 Department of Oral Medicine, Dental and Life Science Institute, School of Dentistry, Pusan National University, Busandaehak-ro 49, Mulgeum-eup, Yangsan 50612, Republic of Korea; oksoomin@pusan.ac.kr (S.-M.O.); jc2wma@pusan.ac.kr (H.-M.J.); drcookie@pusan.ac.kr (S.-H.J.); ahnyongw@pusan.ac.kr (Y.-W.A.); 2 Department of Oral Medicine, Dental Research Institute, School of Dentistry, Pusan National University, Busandaehak-ro 49, Mulgeum-eup, Yangsan 50612, Republic of Korea; 3 Department of Periodontology, Dental Research Institute, Pusan National University Dental Hospital, Yangsan 50612, Republic of Korea; joojy@pusan.ac.kr; 4 Department of Oral Microbiology, School of Dentistry, Pusan National University, Yangsan 50612, Republic of Korea; pajuha2518@pusan.ac.kr; 5 Dental Research Institute, BK21 PLUS Project, School of Dentistry, Pusan National University, Yangsan 50612, Republic of Korea; 6 Department of Oral Microbiology, School of Dentistry, Chonnam National University, Gwangju 61186, Republic of Korea; gji0307@naver.com (S.Y.K.); jchung@chonnam.ac.kr (J.C.)

**Keywords:** oral lichen planus, supragingival plaque, oral microbiome, microbial dysbiosis, microbial interaction network, 16S rRNA gene sequencing

## Abstract

**Background/Objective**: Oral lichen planus (OLP) is a chronic inflammatory disorder of the oral mucosa with unclear etiology. Increasing evidence implicates oral microbial dysbiosis in its pathogenesis, but little is known about supragingival plaque communities in relation to clinical subtypes. This cross-sectional case–control study aimed to characterize the supragingival plaque microbiota and microbial interaction networks in erosive OLP (E-OLP), non-erosive OLP (NE-OLP), and healthy controls (HCs), to elucidate microbial patterns associated with disease severity. **Methods**: Supragingival plaque samples were collected from 90 participants (30 per group) and analyzed using 16S rRNA gene sequencing. Alpha and beta diversity metrics, differential abundance, and co-occurrence network analyses were performed. **Results**: E-OLP exhibited pronounced dysbiosis, including the enrichment of pro-inflammatory taxa (e.g., *Prevotella*, *Parvimonas*) and depletion of health-associated commensals (e.g., *Rothia*, *Capnocytophaga*). Network analysis revealed the stepwise disintegration of microbial community structure from HC to NE-OLP to E-OLP, with reduced connectivity and increased dominance of pathogenic clusters in E-OLP. These microbial alterations aligned with clinical findings, as E-OLP patients showed significantly higher Reticulation/keratosis, Erythema, and Ulceration (REU) scores for erythema and ulceration compared to NE-OLP. **Conclusions**: Supragingival plaque dysbiosis and ecological disruption are strongly associated with OLP severity and subtype. This study highlights the utility of plaque-based microbial profiling in capturing lesion-proximal dysbiotic signals, which may complement mucosal and salivary analyses in future diagnostic frameworks. Multi-omics approaches incorporating fungal, viral, and metabolic profiling are warranted to fully elucidate host–microbe interactions in OLP.

## 1. Introduction

In recent years, advances in next-generation sequencing (NGS) technology have revolutionized our understanding of the human microbiome and its associations with both systemic and localized diseases [1]. The oral cavity, as the entry point to the digestive and respiratory tracts, supports a complex and dynamic microbial ecosystem that is critical for maintaining both mucosal and systemic health [2]. Disruptions in this microbial balance—termed dysbiosis—are increasingly recognized as contributing factors to a range of oral conditions, including periodontitis, dental caries, and mucosal diseases, as well as to systemic disorders [3].

Oral lichen planus (OLP) is a chronic, immune-mediated inflammatory disease that primarily affects the oral mucosa. It is most commonly diagnosed in middle-aged to older adults, particularly women, and is characterized by a T-cell-mediated response to as-yet-unidentified antigens [4]. Although factors such as systemic medications, viral infections, and dental restorative materials have been implicated in OLP pathogenesis, the precise mechanisms and triggers remain elusive. This uncertainty has sparked growing interest in the potential role of the oral microbiome in OLP development and progression [5].

Numerous recent studies have highlighted a bidirectional relationship between oral health and systemic mucocutaneous disorders, including OLP. It is increasingly recognized that poor oral health may exacerbate mucosal inflammation, while conversely, chronic inflammatory mucocutaneous conditions can negatively impact oral microbial balance and hygiene behaviors [6,7,8]. These interactions underscore the relevance of investigating oral microbiota not only as a potential contributor to disease pathogenesis but also as a target for adjunctive management strategies in patients with OLP. Thus, a detailed understanding of the plaque-associated microbial landscape in oral lichen planus may provide valuable insights into the complex host–microbe interactions underlying disease severity and progression.

Clinically, OLP presents with a spectrum of subtypes—including reticular, plaque-like, atrophic, papular, erosive, and bullous forms—with non-erosive (NE) and erosive (E) types being the most clinically relevant. NE-OLP typically appears as asymptomatic white striations or plaques, often requiring only observation, while E-OLP is marked by mucosal ulceration, pain, and bleeding, which can impair oral hygiene and increase the risk of secondary infection and malignant transformation [4]. Immunologically, E-OLP is associated with heightened Th17 cell activity and increased p53 expression, indicating a more aggressive disease phenotype [9,10].

Despite extensive research into the clinical and immunological differences between E-OLP and NE-OLP, the contribution of the oral microbiome—particularly supragingival plaque communities—to these distinctions remains poorly understood. Recent studies suggest that microbiome-derived pro-inflammatory stimuli, ecological imbalance, and interaction network collapse may play pivotal roles in amplifying mucosal immune responses and influencing OLP phenotypic severity. Previous microbiome studies in OLP have largely relied on saliva or buccal swab samples, often with small cohorts and inconsistent methodologies, limiting the reproducibility and generalizability of their findings [11]. Notably, few investigations have focused on supragingival plaque, which serves as a primary reservoir for oral bacteria implicated in both local inflammation and systemic immune activation [12,13].

Supragingival plaque, a dynamic biofilm located on the tooth surface above the gingival margin, plays a pivotal role in maintaining oral microbial homeostasis and initiating host immune responses [14]. Unlike saliva or mucosal swabs, plaque provides a stable ecological niche where microbial communities directly interact with the hard and soft tissues of the oral cavity [15]. Recent evidence suggests that the composition and structure of the supragingival microbiota may be more reflective of site-specific inflammatory conditions, such as those observed in oral lichen planus (OLP), particularly due to its proximity to lesion sites [16]. Moreover, changes in supragingival plaque microbiota may not only mirror disease severity but also contribute actively to mucosal breakdown through the release of microbial metabolites, enzymes, and inflammatory mediators [17,18]. Specifically, plaque enables more-reproducible sampling and hosts site-specific bacterial communities, such as *Actinomyces* and *Capnocytophaga*, which are implicated in mucosal inflammation and immune responses [19]. This ecological distinctiveness highlights the relevance of supragingival plaque as a representative sampling matrix for assessing microbial dysbiosis in oral lichen planus (OLP). Analyzing the plaque microbiome enables localized evaluation of dysbiotic shifts and microbial interaction networks associated with distinct clinical subtypes of OLP, offering more targeted insights into disease pathogenesis than alternative oral sampling methods. Given that mucosal inflammation in OLP can influence the adjacent dental biofilm, plaque samples were collected from tooth surfaces anatomically adjacent to representative lesion sites.

To address these gaps, we aimed to comprehensively characterize and compare the supragingival plaque microbiome in patients with E-OLP, NE-OLP, and healthy controls (HCs) using 16S rRNA gene-based NGS. By identifying microbial taxa associated with disease presence and severity, and by examining microbial community structures and interaction networks, this study seeks to provide novel insights into the microbial dysbiosis underlying OLP pathogenesis. Therefore, an integrative analysis encompassing microbial composition, co-occurrence networks, and inflammation-associated dysbiosis is warranted to elucidate the mechanisms underlying OLP subtype differentiation and to inform future microbiome-based therapeutic strategies. To the best of our knowledge, this is the first study to integrate microbial composition, co-occurrence networks, and plaque-specific dysbiosis across clinical subtypes of oral lichen planus.

## 2. Materials and Methods

### 2.1. Study Population and Sample Collection

This observational study was designed as a cross-sectional case–control investigation comparing supragingival plaque microbiota across patients with erosive OLP, non-erosive OLP, and healthy controls. This study was approved by the Institutional Review Board of Pusan National University Dental Hospital (IRB No. 2024-09-009-004; date: 29 October 2024) and conducted in accordance with the Declaration of Helsinki [20]. All participants provided written informed consent prior to enrollment. A total of 90 participants were enrolled: 60 patients with oral lichen planus (OLP)—30 erosive (E-OLP) and 30 non-erosive (NE-OLP)—and 30 healthy controls (HCs). Participants were recruited from the Department of Oral Medicine at Pusan National University Dental Hospital (Yangsan, South Korea). Inclusion criteria for OLP patients included clinically and histopathologically confirmed diagnoses [18,19]. Healthy controls had no history of oral mucosal disease or systemic conditions known to influence the oral microbiome. Exclusion criteria included use of systemic antibiotics, corticosteroids, statins, or immunosuppressants within 3 months of sampling. Additionally, participants who had used mouthwash within 7 days prior to sampling were excluded to minimize short-term microbiota alterations [21,22]. The clinical severity of OLP was evaluated using the Reticulation/keratosis, Erythema, and Ulceration (REU) scoring system [23].

To control for dietary or hygiene-related variability, participants were instructed to abstain from eating, drinking, or oral hygiene practices for at least 2 h before plaque collection. Supragingival plaque was collected from the buccal surfaces of the teeth (#36–37 or #46–47), which were directly adjacent to the clinical lesions, including those with erosion or Wickham striae. If unilateral lesions were present, plaque was collected from the side closest to the affected mucosa. This sampling strategy was designed to ensure that the plaque microbiota represented communities in anatomical proximity to the mucosal pathology. Plaque was gently collected from non-carious tooth surfaces using sterile curettes and immediately stored at −80 °C in the Biobank of Pusan National University Dental Hospital (Korea Biobank Network: KBN4_A04) [24]. All supragingival plaque samples were collected by a single experienced oral medicine specialist using a standardized sampling protocol to ensure consistency across participants.

### 2.2. Genomic DNA Extraction and 16S rRNA Gene Sequencing

Genomic DNA was isolated from supragingival plaque samples using the Gram-positive DNA purification kit (Lucigen, Biosearch Technology, Novato, CA, USA), following the manufacturer’s guidelines. For amplification of the 16S rRNA V1–V2 regions [25], barcoded fusion primers (27F: 5′-AGA GTT TGA TYM TGG CTC AG-3′ and 338R: 5′-TGC TGC CTC CCG TAG RAG T-3′) were utilized according to the Illumina 16S Metagenomic Sequencing Library protocols. DNA concentrations and purity were assessed using both the PicoGreen assay and NanoDrop ND-1000 spectrophotometry (Thermo Fisher Scientific, MA, USA). DNA samples were stored at −80 °C until sequencing. The pooled amplicon libraries, normalized to equimolar concentrations, underwent paired-end sequencing via the Illumina NovaSeq platform (San Diego, CA, USA).

### 2.3. Bioinformatic Analysis, Statistical Analysis, and Visualization

Sequence data were analyzed with QIIME2 (version 2024.10) [26], employing its core plugins for microbiome analysis. Alpha diversity metrics included the Chao1 richness estimator and Shannon’s diversity index. Beta diversity was evaluated using Bray–Curtis distances with principal coordinate analysis (PCoA) performed via the vegan package (v2.3-0) in R (v3.2.1). Statistical significance for diversity comparisons was assessed using the Kruskal–Wallis test for alpha diversity and PERMANOVA for beta diversity. Taxonomic classification was performed through a pre-trained Naive Bayes classifier against the Human Oral Microbiome Database (eHOMD, version 15.1) [27]. Differentially abundant taxa among the E-OLP, NE-OLP, and HC groups were identified using linear discriminant analysis effect size (LEfSe) [28], applying default parameters and Kruskal–Wallis testing. To explore microbial co-occurrence patterns, network analysis was conducted with Sparse Correlations for Compositional data (SparCC) [29], supplemented by Spearman correlation to determine statistical associations. Networks were visualized using Cytoscape (version 3.9.1, San Diego, CA, USA) [30], where nodes represented taxa and edges indicated correlations.

### 2.4. Data Availability Statement

The raw sequencing data were deposited in NCBI GenBank under BioProject ID PRJEB90477.

## 3. Results

### 3.1. Patient Characterization

The demographic and clinical characteristics of the participants are summarized in Table 1. All three groups had a mean age of over 55 years, and there were no significant differences in age or sex among the groups (*p* > 0.05). The numeric rating scale (NRS), representing the subjective pain scale of the patients, did not differ significantly between the E-OLP and NE-OLP groups, although the E-OLP score was slightly higher. All subjects had more than 20 residual teeth, and there were no differences in the number of teeth among the groups (*p* > 0.05). To evaluate lesion severity more specifically, the REU scores were assessed and compared between the two subtypes (Table 2) [23]. All patients in the E-OLP group presented gingival erosive lesions, observed in combination with erosive lesions in other areas. NE-OLP was scored based on the presence of reticular or plaque forms in the gingiva or other sites. Participants with recent antibiotic or immunosuppressive drug use were excluded from the study. Among the study participants, a history of smoking was reported by one individual in the E-OLP group and two individuals in the NE-OLP group. Alcohol consumption was reported by one participant in the HC group, two in the E-OLP group, and one in the NE-OLP group. Diabetes was present in four participants in the HC group, two in the E-OLP group, and five in the NE-OLP group. All diabetic participants were on medication and had well-controlled blood glucose levels. All participants reported performing oral hygiene practices at least three times per day, and none exhibited clinical signs of periodontal disease. Comprehensive periodontal screening confirmed the absence of active periodontal disease across all groups. In healthy controls (HCs), fewer than 10% of sites exhibited bleeding on probing, and the full-mouth plaque index was below 20%. Similar periodontal status was observed in both the E-OLP and NE-OLP groups, indicating a comparable level of oral hygiene and periodontal health among study participants.

### 3.2. Microbial Diversity in Supragingival Plaque

**Figure 1:**
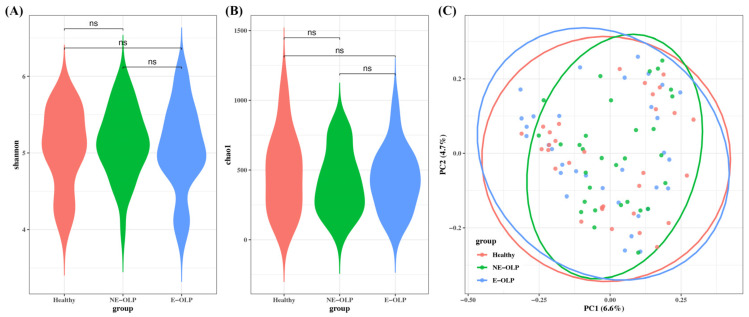
Microbial diversity analysis of supragingival plaque samples in healthy controls, NE-OLP, and E-OLP groups. (**A**) Violin plot of Shannon diversity index showing alpha diversity (species evenness and richness) across groups. (**B**) Violin plot of Chao1 index representing estimated species richness. (**C**) Principal coordinate analysis (PCoA) based on Bray–Curtis distances demonstrating beta diversity and overall community structure. No statistically significant differences observed in alpha or beta diversity among three groups (ns, not significant). Color coding: red—healthy controls; green—non-erosive oral lichen planus (NE-OLP); blue—erosive oral lichen planus (E-OLP).

Alpha diversity, assessed by Shannon and Chao1 indices, did not show statistically significant differences among the healthy control (HC), non-erosive OLP (NE-OLP), and erosive OLP (E-OLP) groups (Figure 1A,B). This indicates that overall species richness and evenness were comparable across the three groups. Principal coordinate analysis (PCoA) based on Bray–Curtis distances also demonstrated no clear separation among the groups, suggesting similar overall community structures (Figure 1C).

### 3.3. Taxonomic Composition and Relative Abundance

**Figure 2:**
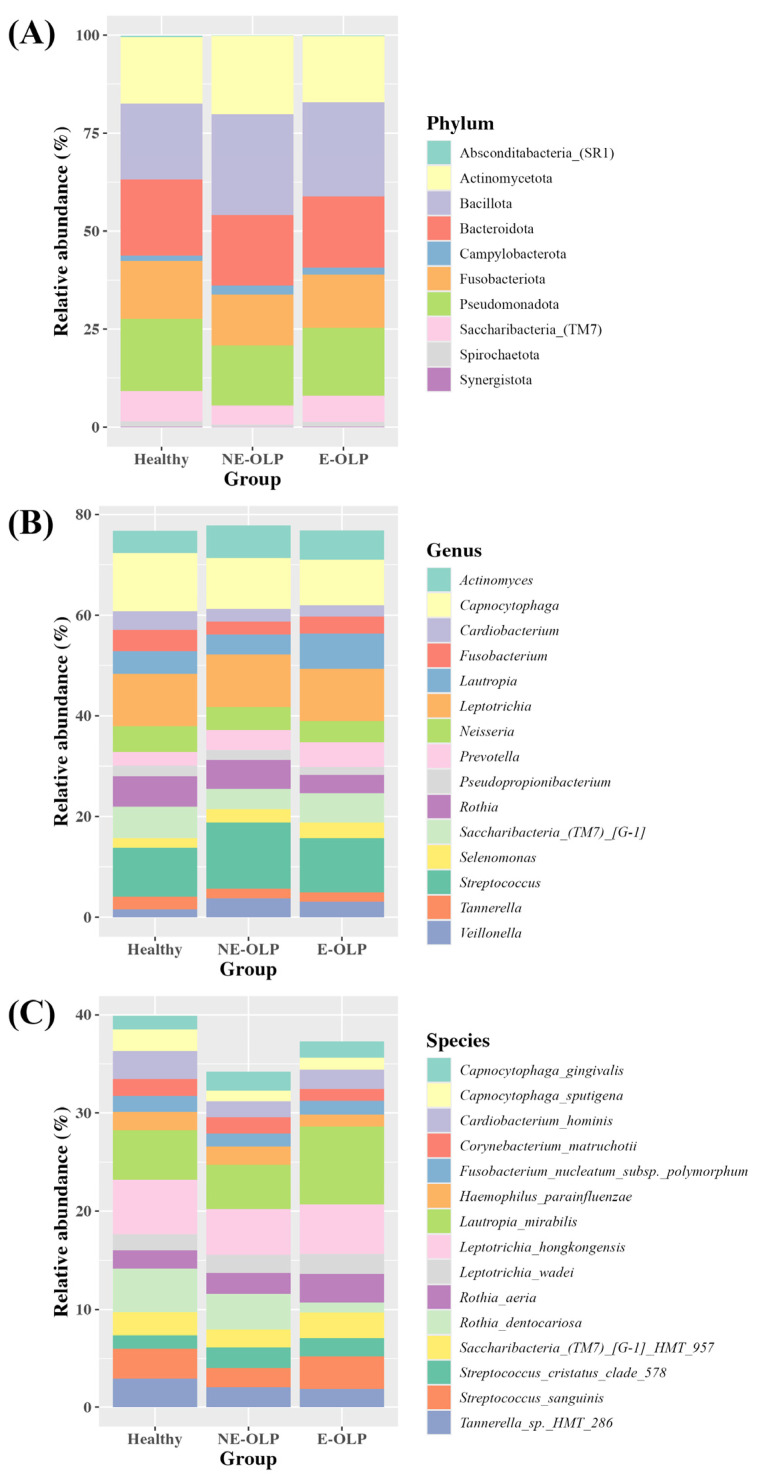
Relative abundance of supragingival plaque microbiota at different taxonomic levels among study groups. Stacked bar plot showing relative abundance (%) of bacterial at (**A**) phylum level, (**B**) genus level, (**C**) species level. Taxa with top 15 highest relative abundance presented in legend. NE-OLP: non-erosive OLP; E-OLP: erosive OLP (E-OLP).

At the phylum, genus, and species levels, the composition of the supragingival plaque microbiome was broadly similar among groups, but notable differences in the relative abundance of specific taxa were observed (Figure 2A–C). At the phylum level, six dominant phyla—Bacillota, Actinomycetota, Bacteroidota, Pseudomonadota, Fusobacteriota, and Saccharibacteria—collectively accounted for the majority of microbial composition across all groups. Among them, Bacillota was most abundant in the NE-OLP group (25.7%), followed by E-OLP (24.1%) and HCs (19.3%). Actinomycetota was also more prevalent in NE-OLP (20.1%) and the HCs (16.9%) compared to E-OLP (16.9%). The proportions of Bacteroidota and Pseudomonadota remained relatively stable across the three groups. Notably, Saccharibacteria—a phylum frequently associated with oral health—was highest in the HCs (3.9%) and progressively decreased in NE-OLP (2.5%) and E-OLP (1.5%), suggesting a potential link between the loss of this taxon and disease progression (Figure 2A).

At the genus level, a total of 223 genera were identified, with *Streptococcus, Capnocytophaga, Leptotrichia, Lautropia,* and *Actinomyces* being the most dominant. *Streptococcus* exhibited the highest abundance in NE-OLP (13.2%), followed by E-OLP (10.8%) and the HCs (9.7%), possibly reflecting early microbial responses to subclinical mucosal inflammation in the non-erosive type. Conversely, *Capnocytophaga* showed a decreasing trend from the HCs (11.6%) to NE-OLP (10.1%) and E-OLP (9.2%), which may indicate a reduction in health-associated species in the diseased states. *Lautropia,* previously linked to inflamed mucosal conditions, was more abundant in E-OLP (7.0%) than in NE-OLP (3.9%) and the HCs (4.4%) (Figure 2B).

At the species level, several taxa displayed distinct abundance patterns among the groups. *Lautropia mirabilis* was particularly enriched in the E-OLP group (7.9%), compared to NE-OLP (4.5%) and the HCs (5.0%). In contrast, *Rothia dentocariosa,* an oral commensal with known anti-inflammatory properties, was markedly depleted in E-OLP (1.0%) relative to the HCs (4.4%) and NE-OLP (3.6%), suggesting its potential protective role in oral mucosal health. *Streptococcus sanguinis* displayed similar abundances in the HCs (3.1%) and E-OLP (3.3%), but was lower in NE-OLP (2.0%). Interestingly, *Rothia aeria* showed a progressive increase across the three groups: 1.9% in HC, 2.1% in NE-OLP, and 2.9% in E-OLP (Figure 2C).

Overall, these findings support the notion that the transition from healthy to diseased oral mucosa is marked by a relative loss of beneficial commensals and an enrichment of potentially pathogenic or pro-inflammatory species. In particular, the depletion of *Rothia dentocariosa* and the enrichment of *Lautropia mirabilis* in E-OLP suggest that supragingival microbial dysbiosis may play a contributory role in OLP severity.

### 3.4. Differential Abundance of Supragingival Plaque Microbiota Among OLP Subtypes and Healthy Controls

**Figure 3:**
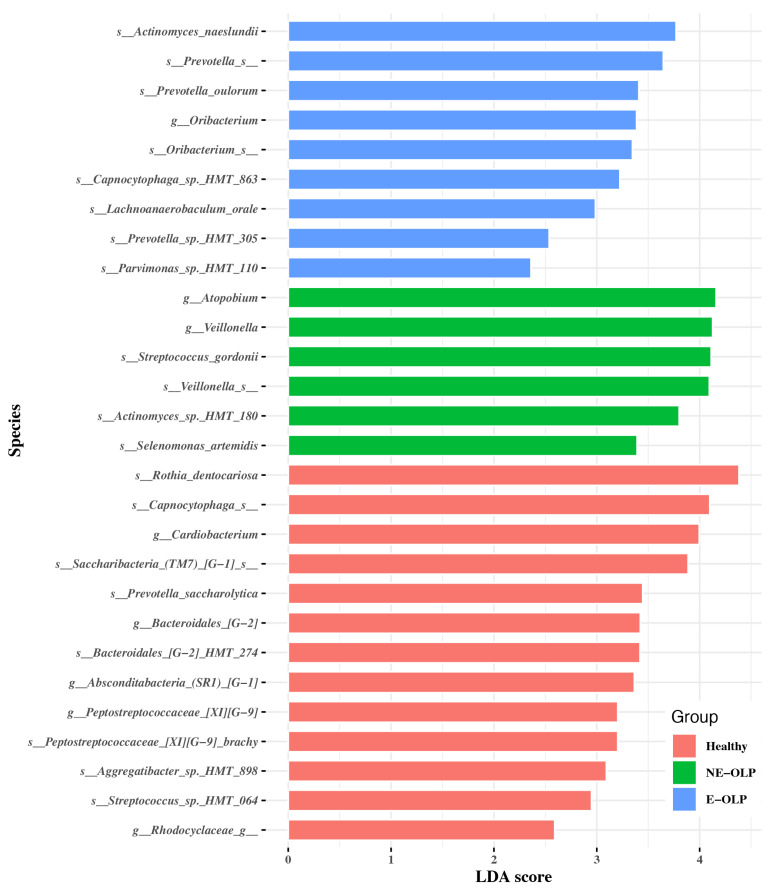
LEfSe analysis showing differentially abundant bacterial taxa among HC, NE-OLP, and E-OLP groups with LDA scores.

LEfSe analysis revealed distinct differences in the composition of the supragingival plaque microbiome among the three groups (Figure 3). In the erosive OLP (E-OLP) group, the following taxa were significantly enriched: *Actinomyces naeslundii*, *Prevotella* spp., *Prevotella oulorum*, *Oribacterium Capnocytophaga* sp. *HMT 863, Lachnoanaerobaculum orale*, *Prevotella* sp. *HMT 305*, and *Parvimonas* sp. *HMT 110*. These bacteria were characteristically increased in the supragingival plaque of patients with erosive OLP. In contrast, the non-erosive OLP (NE-OLP) group exhibited higher abundances of *Atopobium*, *Veillonella*, *Streptococcus gordoni, Veillonella* spp., *Actinomyces* sp. *HMT 180*, and *Selenomonas artemidis*, reflecting a relatively stable and balanced microbial community compared to the erosive subtype. The healthy control (HC) group was significantly enriched with *Rothia dentocariosa*, *Capnocytophaga* spp., *Cardiobacterium*, *Saccharibacteria (TM7) [G-1]* sp., *Prevotella saccharolytica*, *Bacteroidales [G-2] HMT. 274*, *Absconditabacteria (SR1) [G-1]*, *Peptostreptococcaceae [XI] [G-9] brachy*, *Aggregatibacter* sp. *HMT 898, Streptococcus* sp. *HMT 064*, and *Rhodocyclaceae genus*. These taxa were predominantly found in healthy oral environments, highlighting clear differences in microbial composition associated with clinical subtypes of OLP and health.

### 3.5. Differential Abundance of Key Bacterial Species Among Healthy Control, NE-OLP, and E-OLP Groups

**Figure 4:**
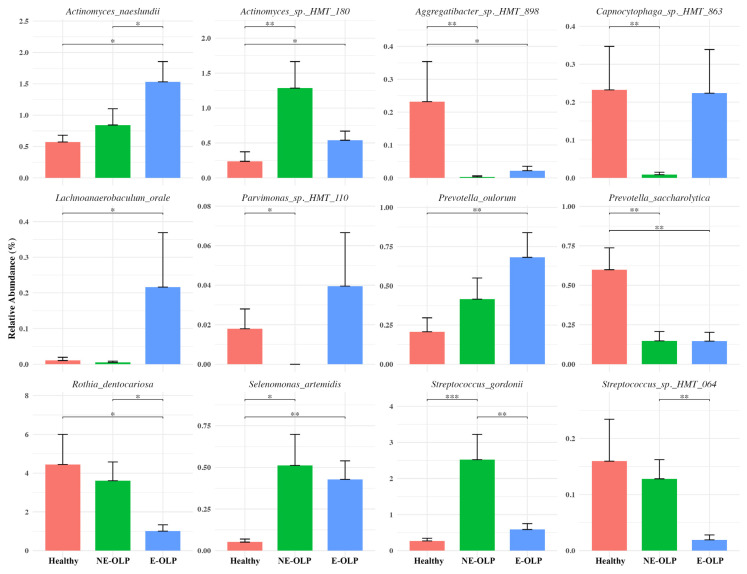
Relative abundance of representative bacterial species in supragingival plaque among healthy controls (red), non-erosive OLP (NE-OLP, green), and erosive OLP (E-OLP, blue). Bar plots show mean ± standard deviation of relative abundance (%) for each species. *: *p* < 0.05; **: *p* < 0.01; ***: *p* < 0.001.

To further validate the statistical difference, the relative abundances of significant taxa determined by LEfSe were compared (Figure 4). The species *Actinomyces naeslundii* showed a progressive increase from Healthy to NE-OLP and was highest in E-OLP, with statistically significant differences between all groups. Similarly, *Actinomyces sp. HMT 180* was most abundant in NE-OLP, significantly higher than in both the Healthy and E-OLP groups. *Aggregatibacter* sp. *HMT 898* and *Capnocytophaga* sp. *HMT 863* were significantly more abundant in healthy controls compared to both OLP groups, with the lowest levels observed in NE-OLP and E-OLP. *Lachnoanaerobaculum orale* and *Parvimonas sp. HMT 110* were enriched in E-OLP compared to the Healthy and NE-OLP groups, indicating a potential association with the erosive subtype. Among *Prevotella* species, *Prevotella oulorum* and *Prevotella saccharolytica* were significantly more abundant in E-OLP than in the Healthy and NE-OLP groups. The commensal species *Rothia dentocariosa* was significantly depleted in E-OLP compared to Healthy and NE-OLP, while *Selenomonas artemidis* showed the highest abundance in NE-OLP, significantly greater than in Healthy and E-OLP. *Streptococcus gordonii* was markedly enriched in NE-OLP, exceeding levels in both the Healthy and E-OLP groups, whereas *Streptococcus* sp. *HMT 064* was most abundant in healthy controls and significantly reduced in the OLP groups. Distinct microbial profiles were observed across clinical subtypes of OLP. E-OLP samples showed an increased relative abundance of potential pathogenic taxa, whereas NE-OLP samples exhibited an intermediate microbial composition enriched with commensal streptococci such as Streptococcus gordonii.

### 3.6. Co-Occurrence Network Analysis Reveals Progressive Disruption of Microbial Interactions in OLP

**Figure 5:**
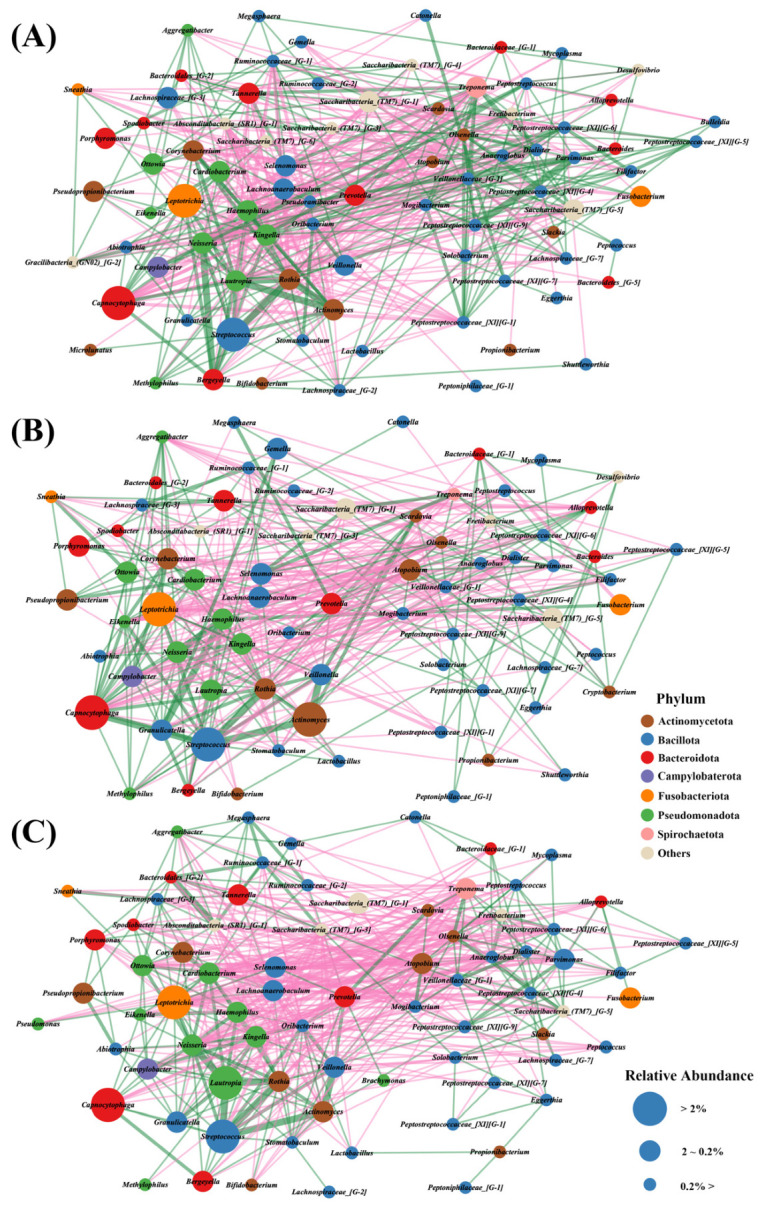
Co-occurrence network analysis of supragingival plaque microbiota in (**A**) the healthy controls, (**B**) non-erosive OLP (NE-OLP), (**C**) erosive OLP (E-OLP). Each node represents a bacterial taxon, colored by phylum, and the size of the node indicates its relative abundance. The edges represent significant correlations between taxa: green lines denote positive associations and pink lines denote negative associations. The healthy control network (**A**) is densely interconnected, reflecting a stable and cooperative microbial community. In NE-OLP (**B**), overall network complexity and connectivity are reduced, with more negative correlations involving potential pathogens. In E-OLP (**C**), the network is further fragmented, with increased interactions among pathogenic taxa and a pronounced loss of connectivity among commensals, indicating the progressive dysbiosis and instability of the microbial community structure with increased disease severity.

Finally, the co-occurrence networks of supragingival plaque microbiota depending on group were analyzed. In the healthy control group, the network is highly complex and densely interconnected, with numerous positive correlations among commensal bacteria such as *Streptococcus*, *Veillonella*, *Rothia*, and *Neisseria.* Microbial co-occurrence network analysis revealed a dense and highly connected network in the healthy controls (Figure 5A). In NE-OLP, network connectivity was reduced, and an increased number of negative correlations involving Porphyromonas and Tannerella were observed (Figure 5B). E-OLP samples showed the greatest disruption in network structure, with reduced connectivity among commensals and increased positive correlations among pathogenic species such as Prevotella intermedia, Treponema socranskii, and Fusobacterium nucleatum, as well as more frequent negative interactions between commensals and pathogens (Figure 5C). This fragmented and modular network structure suggests that the microbial community in E-OLP is less stable and more susceptible to domination by pro-inflammatory and pathogenic species. Overall, these network analyses reveal a progressive breakdown of microbial community stability and connectivity from healthy controls to NE-OLP and E-OLP, highlighting the role of disrupted microbial interactions in the pathogenesis and severity of OLP.

## 4. Discussion

This study compared the supragingival plaque microbiota and microbial interaction networks across erosive oral lichen planus (E-OLP), non-erosive OLP (NE-OLP), and healthy controls (HCs) to elucidate microbial shifts associated with OLP severity and clinical subtypes. By integrating compositional and ecological perspectives, our findings underscore that microbial dysbiosis and inflammation-associated community expansion are closely linked to the pathophysiology and clinical severity of OLP.

Unlike most previous studies [11,13] that have focused on salivary or mucosal microbiomes, this study specifically targeted the supragingival plaque microbiota—an ecologically distinct niche directly involved in oral immune responses and biofilm-associated pathology [12]. Supragingival plaque harbors densely populated microbial communities that interact with the gingival epithelium and contribute to local inflammation through metabolic and structural mechanisms [17,18]. By analyzing plaque-derived microbiota, we captured the microbial signals most proximal to the lesion environment, allowing for more precise associations with epithelial damage and immune dysregulation. Moreover, this plaque-based approach offers unique insight into microbial biofilm stability, ecological succession, and network disruption, which are often masked in salivary or tissue-level analyses. These features make our findings particularly relevant for understanding the site-specific microbial dynamics underlying OLP subtypes. Supragingival plaque was chosen as the sampling matrix because it provides a non-invasive, reproducible site that may reflect microbial shifts associated with mucosal inflammation in OLP. Previous studies suggest that microbial translocation and biofilm-mediated inflammation may extend from plaque to adjacent mucosal lesions, justifying its use in evaluating host–microbiota interactions [3,16]. However, it should be noted that plaque samples may not fully capture the local microbial dynamics at the mucosal surface, particularly within erosive lesions, where tissue-resident or adherent bacteria may differ significantly. Future studies integrating both supragingival and mucosal sampling (e.g., swab or biopsy) could provide a more comprehensive understanding of the spatial heterogeneity of the OLP-associated microbiome.

In E-OLP, there was a notable enrichment of pro-inflammatory bacteria such as *Prevotella* spp., *Actinomyces naeslundii*, and *Parvimonas* spp. which are known to secrete proteases and lipopolysaccharides (LPSs), thereby aggravating epithelial damage and amplifying Th1/Th17 immune responses [5,31]. *Prevotella* has been reported to promote IL-17/IL-23-axis-mediated inflammation [5,31], while *Oribacterium,* another genus enriched in E-OLP, has been implicated in the metabolic reprogramming of the local microenvironment, potentially influencing OLP phenotypic expression [32]. Conversely, HC samples exhibited dominance of health-associated commensals such as *Rothia dentocariosa* and *Capnocytophaga* spp., which are known to inhibit pathogenic colonization via siderophore production and niche competition [33]. The marked depletion of these taxa in OLP, especially in the erosive form, may signify a shift toward an opportunistic and dysbiotic microbial environment.

The NE-OLP group demonstrated a relatively balanced microbial composition enriched with commensals such as *Atopobium*, *Veillonella*, *Streptococcus gordonii*, and *Selenomonas artemidis*, which are commonly found in healthy oral ecosystems and contribute to low-inflammatory mucosal conditions. The high abundance of *S. gordonii* in NE-OLP could be related to epithelial proliferation and the characteristic Wickham’s striae, which defines this OLP subtype histologically [34].

Microbial co-occurrence network analysis further revealed a stepwise breakdown of community architecture from HCs to NE-OLP to E-OLP [5,13]. In HCs, commensals like *Streptococcus*, *Veillonella*, and *Rothia* formed highly interconnected and cooperative networks. However, as disease severity increased, network complexity and connectivity diminished, while antagonistic interactions between commensals and pathogens intensified. In E-OLP, clusters of pathogenic bacteria dominated the network, reflecting ecological instability and a loss of resilience in microbial interactions. This aligns with the clinical assessment, in which E-OLP patients showed significantly higher REU scores for erythema and ulceration compared to NE-OLP patients (Table 2), reinforcing the relevance of microbial imbalance in severe OLP presentations.

Importantly, this study not only confirms findings from previous studies—which identified pathogens like *Porphyromonas* and *Solobacterium* in OLP [5,32]—but also extends them by stratifying microbial profiles and interaction patterns according to clinical subtype. While many earlier studies focused primarily on taxonomic abundance [35], our integration of network analysis offers a deeper understanding of the ecological collapse accompanying severe disease progression.

This study has several limitations. First, it focused exclusively on bacterial communities using 16S rRNA gene sequencing, thereby excluding other key microbial kingdoms such as fungi and viruses. This single-kingdom approach may overlook important interactions within the broader oral microbiome. For instance, recent studies have reported that fungal–bacterial imbalances, particularly *Candida* overgrowth, are implicated in OLP pathogenesis and may promote pathogenic bacterial colonization and secondary infections [13,36]. Additionally, this study did not assess host-derived metabolic profiles, despite emerging evidence suggesting that alterations in lipid and amino acid metabolism are closely associated with microbiome composition in OLP patients [13,16].

Given these limitations, future research would benefit from a multi-omics approach, incorporating internal transcribed spacer sequencing for fungal community profiling, metabolomics to assess host–microbe interactions, and viral metagenomics to explore the potential influence of bacteriophages and eukaryotic viruses on microbial ecology and mucosal inflammation. Additionally, systemic analyses such as Mendelian randomization and oral–gut axis studies could help elucidate the broader microbial contributions to OLP pathogenesis. Importantly, as this study focused solely on supragingival plaque, it may not fully represent the site-specific microbial dynamics at the mucosal surface, where direct host–microbiota interactions occur. Future studies integrating mucosal swabs or biopsies are warranted to capture a more localized and comprehensive view of the lesion-associated microbiota in OLP.

Collectively, these results illustrate that microbial dysbiosis, pathogenic enrichment, and interaction collapse are central to the immunopathogenesis and clinical severity of OLP. This underscores the need for future multi-omics approaches—including metabolomics and mycobiome profiling—and for systems-level investigations of the oral–gut microbial continuum. Microbiome-informed diagnostics and therapeutics targeting both microbial composition and ecological structure may offer novel strategies for managing OLP [37]. These insights may enhance clinical diagnostic precision by enabling the use of microbial biomarkers to distinguish between erosive and non-erosive OLP. Furthermore, understanding the distinct microbial profiles associated with disease severity may guide clinicians in tailoring treatment strategies, particularly in selecting anti-inflammatory or antimicrobial adjuncts for patients exhibiting pathogenic microbial signatures. Ultimately, our findings highlight the potential for integrating oral microbiome profiling into personalized diagnostic and therapeutic frameworks for OLP management.

## 5. Conclusions

This study reveals distinct microbial community structures in supragingival plaque across clinical subtypes of oral lichen planus. Patients with erosive OLP exhibited reduced microbial diversity, increased abundance of pro-inflammatory taxa, and disrupted microbial interaction networks compared to those with non-erosive OLP and healthy controls. These findings suggest that supragingival dysbiosis not only reflects but may actively contribute to disease severity in OLP.

Importantly, the identification of subtype-specific microbial signatures may support the development of microbial biomarkers to enhance diagnostic precision between erosive and non-erosive OLP. Furthermore, understanding the ecological dynamics of the plaque microbiome may inform personalized treatment strategies, including the use of anti-inflammatory or antimicrobial adjuncts tailored to each patient’s microbial profile. These insights underscore the clinical potential of integrating oral microbiome profiling into individualized diagnostic and therapeutic approaches for OLP management.

## Figures and Tables

**Table 1:** Patient characteristics.

Characteristics	Healthy (*n* = 30)	Erosive OLP (*n* = 30)	Non-Erosive OLP (*n* = 30)	*p*-Value
Age (mean ± SD)	56.7 ± 13.8	59.7 ± 9.9	59.6 ± 10.4	0.95172
Male/female	M:7/F:23	M:10/F:20	M:11/F:19	0.51000
NRS	-	1.7 ± 2.3	1.1 ± 1.9	0.71992
No. of teeth (mean ± SD)	27.7 ± 2.2	25.7 ± 3.3	23.4 ± 5.3	0.83455
Cigarette smoking	0 (0%)	1 (3.3%)	2 (6.7%)	-
Alcohol drinking	1 (3.3%)	2 (6.7%)	1 (3.3%)	-
Diabetes	4 (13.3%)	2 (6.7%)	5 (16.7%)	-
Oral hygiene frequency ≥3/day (*n*,%)	30 (100%)	30 (100%)	30 (100%)	-
Clinical signs of periodontitis (*n*, %)	0 (0%)	0 (0%)	0 (0%)	-

**Table 2:** Comparison of Reticulation, Erythema, and Ulceration scores between erosive and non-erosive oral lichen planus groups.

Clinical Component	Erosive Oral Lichen Planus (*n* = 30)	Non-Erosive Oral Lichen Planus (*n* = 30)	*p*-Value
Reticulation/Keratosis (Score ≥ 2)	30 (100.0%)	30 (100.0%)	–
Erythema (Score ≥ 2)	30 (100.0%)	0 (0.0%)	<0.001
Ulceration (Score ≥ 2)	8 (26.7%)	0 (0.0%)	0.005
Mean Total Score ± Standard Deviation	7.6 ± 2.4	2.4 ± 1.3	<0.001

Each clinical component was evaluated using the Reticulation/keratosis, Erythema, and Ulceration (REU) scoring system [23], which assigns a severity score (0–3) to each of the 10 intraoral subsites. The total score reflects the overall clinical severity of oral lichen planus. *p*-values were calculated using Pearson’s chi-square test for categorical variables and an independent-sample *t*-test for the continuous variable (total score).

## Data Availability

The raw sequencing data have been deposited in NCBI GenBank under BioProject ID PRJEB90477.

## Abbreviations

The following abbreviations are used in this manuscript:

OLP	Oral lichen planus
E-OLP	Erosive oral lichen planus
NE-OLP	Non-erosive oral lichen planus
HC	Healthy control
REU	Reticulation, erythema, and ulceration
NGS	Next-generation sequencing
LEfSe	Linear discriminant analysis effect size
SRA	Sequence read archive
LDA	Linear discriminant analysis
OTU	Operational taxonomic unit
PCR	Polymerase chain reaction

## References

[1] (2023). Nature Outlook: The Human Microbiome. Nature.

[2] Chaturvedi A.K., Vogtmann E., Shi J., Yano Y., Blaser M.J., Bokulich N.A., Caporaso J.G., Gillison M.L., Graubard B.I., Hua X. (2025). Oral Microbiome Profile of the US Population. JAMA Netw. Open.

[3] Hajishengallis G. (2015). Periodontitis: From microbial immune subversion to systemic inflammation. Nat. Rev. Immunol..

[4] Wang H., Zhang L., Shi Y., Wang Y., Yang J., Wang J. (2022). The Role of T Cells in Oral Lichen Planus: Pathogenesis and Clinical Implications. Front. Immunol..

[5] Wang K., Zhang H., Zhang H., Wang L., Yao Y., Zhang Y. (2016). Preliminary analysis of salivary microbiome and their potential roles in oral lichen planus. Sci. Rep..

[6] Cassol-Spanemberg J., Rodríguez-de Rivera-Campillo M.E., Otero-Rey E.M., Estrugo-Devesa A., Jané-Salas E., López-López J. (2018). Oral lichen planus and its relationship with systemic diseases. A review of evidence. J. Clin. Exp. Dent..

[7] Li Y., Wang K., Zhang B., Zhang W., Chen J., Wu L., Ren B., He J., Shen X., Van Nostrand J.D. (2019). Salivary mycobiome dysbiosis and its potential impact on bacteriome shifts and host immunity in oral lichen planus. Int. J. Oral Sci..

[8] Botelho J., Mascarenhas P., Viana J., Proença L., Orlandi M., Mendes J.J., Machado V. (2022). An umbrella review of the evidence linking oral health and systemic noncommunicable diseases. Nat. Commun..

[9] Sugerman P.B., Savage N.W., Walsh L.J., Zhao Z.Z., Zhou X.J., Khan A., Seymour G.J., Bigby M. (2002). The pathogenesis of oral lichen planus. Crit. Rev. Oral Biol. Med..

[10] Zhou T., Liu Y., Li Y., Zhou Z., Wang X., Li X., Wang Y., Zhou X. (2019). p53 expression in oral lichen planus and its association with clinical characteristics: A retrospective study. J. Oral Pathol. Med..

[11] Cheng Y.S., Gould A., Kurago Z.B., Fantasia J.E., Muller S., Park H., Diehl S.R., Reichart P.A., Wright J.M., Jordan R.C.K. (2020). Methodological challenges in studying the oral microbiome in oral lichen planus. J. Oral Microbiol..

[12] Abusleme L., Dupuy A.K., Dutzan N., Silva N., Burleson J.A., Strausbaugh L.D., Gamonal J., Diaz P.I. (2013). The subgingival microbiome in health and periodontitis and its relationship with community biomass and inflammation. ISME J..

[13] Beibei L., Mengying W., Xiao H., Yuzi J., Lijin M., Ke Z., Shengjie Y., Li L. (2024). Dysbiosis and interactions of the mycobiome and bacteriome in mucosal lesions of erosive and non-erosive oral lichen planus patients. J. Oral Microbiol..

[14] Marsh P.D. (2006). Dental plaque as a biofilm and a microbial community—Implications for health and disease. BMC Oral Health.

[15] Simon-Soro A., Mira A. (2015). Solving the etiology of dental caries. Trends Microbiol..

[16] Zhou Y., Liu W., Huang X., Shi X., Zhao G., Yang Z. (2022). Microbiome landscape of lesions and adjacent normal mucosal sites in oral lichen planus. Front. Microbiol..

[17] Lamont R.J., Koo H., Hajishengallis G. (2018). The oral microbiota: Dynamic communities and host interactions. Nat. Rev. Microbiol..

[18] Alejandro Borrego-Ruiz J.J. (2025). Borrego. Human oral microbiome and its influence on mental health and brain disorders. AIMS Microbiol..

[19] Kilian M., Chapple I.L.C., Hannig M., Marsh P.D., Meuric V., Pedersen A.M.L., Tonetti M.S., Wade W.G., Zaura E. (2016). The oral microbiome—An update for oral healthcare professionals. Br. Dent. J..

[20] World Medical Association (2013). World Medical Association Declaration of Helsinki: Ethical Principles for Medical Research Involving Human Subjects. JAMA.

[21] van der Meij E.H., van der Waal I. (2003). Lichen planus and lichenoid lesions of the oral mucosa: A diagnostic challenge. Oral Surg. Oral Med. Oral Pathol. Oral Radiol. Endod..

[22] González-Moles M.Á., Scully C., Gil-Montoya J.A., Ruiz-Ávila I., Plaza-Campillo J.J. (2019). World Workshop on Oral Medicine VII: Biomarkers of Oral Lichen Planus. Oral Dis..

[23] Thongprasom K., Luangjarmekorn L., Sererat T., Taweesap W. (1992). A scoring system for the quantitative assessment of oral lichen planus. J. Oral Pathol. Med..

[24] Korea Biobank Network (2024). Standard Operating Procedures for Biobanking.

[25] Klindworth A., Pruesse E., Schweer T., Peplies J., Quast C., Horn M., Glöckner F.O. (2013). Evaluation of general 16S ribosomal RNA gene PCR primers for classical and next-generation sequencing-based diversity studies. Nucleic Acids Res..

[26] Bolyen E., Rideout J.R., Dillon M.R., Bokulich N.A., Abnet C.C., Al-Ghalith G.A., Alexander H., Alm E.J., Arumugam M., Asnicar F. (2019). Reproducible, interactive, scalable and extensible microbiome data science using QIIME 2. Nat. Biotechnol..

[27] Escapa I.H., Chen T., Huang Y., Gajare P., Dewhirst F.E., Lemon K.P. (2018). New Insights into Human Nostril Microbiome Using the Expanded Human Oral Microbiome Database (eHOMD). NPJ Biofilms Microbiomes.

[28] Segata N., Izard J., Waldron L., Gevers D., Miropolsky L., Garrett W.S., Huttenhower C. (2011). Metagenomic biomarker discovery and explanation. Genome Biol..

[29] Friedman J., Alm E.J. (2012). Inferring correlation networks from genomic survey data. PLoS Comput. Biol..

[30] Shannon P., Markiel A., Ozier O., Baliga N.S., Wang J.T., Ramage D., Amin N., Schwikowski B., Ideker T. (2003). Cytoscape: A software environment for integrated models of biomolecular interaction networks. Genome Res..

[31] Nouraddini N., Dugashvili G. (2025). Oral Microbiome and Its Role in Oral Lichen Planus Development: A Literature Review. Eur. Sci. J..

[32] Beibei L., Mengying W., Xiao H., Yuzi J., Lijin M., Ke Z., Shengjie Y., Li L. (2024). Alterations of oral microbiome and metabolic signatures and their association with oral lichen planus. J. Oral Microbiol..

[33] Escapa I.H., Chen T., Huang Y., Gajare P., Dewhirst F.E., Lemon K.P. (2020). Commensal Oral *Rothia mucilaginosa* Produces Enterobactin, a Siderophore with Potential Beneficial Effects. mSystems.

[34] Zhou X., Wang Y., Wang X., Li Y., Li X., Zhou Z., Liu Y., Wang Y., Zhou X. (2020). *Streptococcus gordonii* modulates epithelial cell proliferation and immune responses in oral lichen planus. Front. Cell. Infect. Microbiol..

[35] Jung W., Jang S. (2022). Oral Microbiome Research on Oral Lichen Planus: Current Findings and Perspectives. Biology.

[36] Salazar C.R., Sun J., Li Y., Francois F., Corby P., Diaz P., Wang X. (2019). Oral mycobiome and lichen planus: A cross-sectional study. J. Dent. Res..

[37] Smith J., Lee H. (2025). Probiotic and prebiotic applications in oral mucosal lesions: A review. npj Biofilms Microbiomes.

